# Association of physical activity, screen time and sleep with substance use in children and adolescents: a large sample cross-sectional study

**DOI:** 10.3389/fpubh.2024.1432710

**Published:** 2024-10-17

**Authors:** Huadong Su, Dongye Lyu, Ke Huang, Jin Yan

**Affiliations:** ^1^Faculty of Medicine, Veterinary Medicine, and Life Sciences, University of Glasgow, Glasgow, United Kingdom; ^2^College of Education Sciences, The Hong Kong University of Science and Technology, Guangzhou, Guangzhou, China; ^3^School of Psychology, Shenzhen University, Shenzhen, China; ^4^School of Physical Education and Sports Science, Soochow University, Suzhou, China

**Keywords:** physical activity, screen time, sleep, substance use, China

## Abstract

**Background:**

24-h movement guidelines (24-HMG) play an important role in various demographics such as early years, children, youth, and the older adult. Nevertheless, most existing research exploring the links between socioeconomic factors, dietary intake, and substance use with sleep patterns, physical activity (PA), and sedentary behavior (SB) has been conducted in high-income Western countries.

**Purpose:**

Hence, this study seeks to investigate the relationship between adherence to the 24-HMG and smoking and alcohol use behaviors among children and adolescents in China.

**Methods:**

A comprehensive survey, in collaboration with the Municipal Education Commission, was carried out across primary and middle schools in Shenzhen, China. Sleep duration was gauged using the Pittsburgh Sleep Quality Index (PSQI), screen time was assessed with items adapted from the Health Behavior of School-aged Children (HBSC) survey, and PA was measured using a single item adapted from the HBSC survey. Results were presented as odds ratios (ORs) with 95% confidence intervals (CIs), considering *p*-values below 0.05 as statistically significant.

**Results:**

Among the sample, 51.9% were boys and 48% were girls. Those who did not meet any guidelines had a higher probability of smoking (OR = 1.62 [95% CI: 1.03, 2.56], *p* = 0.037) among children and adolescents. Conversely, meeting one (OR = 0.94 [95% CI: 0.61, 1.52], *p* = 0.874) or two guidelines (OR = 0.84 [95% CI: 0.52, 1.34], *p* = 0.459) showed no significant impact. The data displayed an inverse correlation between the number of guidelines adhered to and the likelihood of alcohol use among children and adolescents: none (OR = 2.07, *p* < 0.001), one guideline (OR = 1.40, *p* = 0.006), and two guidelines (OR = 1.22, *p* = 0.106).

**Conclusion:**

Not meeting guidelines elevates smoking and alcohol use risks in children and adolescents, whereas following more guidelines lowers these risks, highlighting the importance of guideline adherence in reducing substance use.

## 1 Introduction

Regular participation in physical activity (PA) elicits a multitude of health benefits, encompassing enhanced muscular and cardiorespiratory fitness, facilitation of optimal bone mineralization (1–4), augmentation of subjective well-being (e.g., self-perception, self-esteem, and affective state), and mitigation of psychopathological symptoms (e.g., depression, anxiety, and stress) (5–8). Furthermore, PA is also correlated with heightened cognitive functioning and academic performance (2, 9–11). The World Health Organization (WHO) recommends that children and adolescents engage in at least 60 min of moderate to vigorous-intensity physical activity (MVPA) daily. (12, 13). PA during children and adolescence creates complete health for the youth (4, 14, 15), and also improves cardiovascular health, fitness levels, and the prevention of chronic illnesses (16–18). The positive effect is also extended to the mental realm, as PA serves to reduce symptoms of anxiety and depression. They are also psychologically beneficial, as they improve mood, self-esteem, and general emotional well-being (19). Additionally, sports and other PA are significant for the development of vital life skills such as teamwork, discipline, and perseverance (20, 21). These experiences are very formative and do not affect just health outcomes at that moment, but make a foundation for a healthy active lifestyle in the future (22). Hence, the importance of promoting and encouraging PA amongst youth in fostering their overall well-being cannot be underestimated.

Recent epidemiological studies in PA and related disciplines have increasingly acknowledged the significance of 24-h movement behaviors (2, 23–26). This encompasses PA, sedentary behavior (SB), and sleep, which are recognized as interdependent, wherein alterations in one behavior may impact the others (27, 28). Incorporating an integrated approach to these behaviors in health-related research is paramount. Such an approach facilitates a comprehensive analysis, yielding deeper insights into health outcomes and behavioral modifications. The growing emphasis on these behaviors in research is attributed to the myriad of health benefits associated with each behavior when adhered to at recommended levels.

Intervention experiments have been undertaken with the aim of augmenting PA, mitigating sedentary behavior (SB), and fostering adequate sleep (29–31). Researchers advocate for a minimum of 60 min of MVPA per day, limiting screen time to no more than 2 h daily, and ensuring adequate sleep across all age groups (26, 32). Since 2015, countries including Canada, Australia, New Zealand, and South Africa have released tailored (24-HMG) for various demographics such as early years, children, youth, and the older adult. The 24-HMG for children and adolescents provide a comprehensive framework that balances various behaviors over a 24-h period, recognizing that health is shaped by the cumulative effects of daily routines. These guidelines highlight the significance of incorporating movement behaviors consistently throughout the day to enhance health outcomes (27, 33, 34).

In addition to the association between the volume of PA and SB, research has unveiled the significance of considering a range of sociodemographic, dietary, and substance use factors that might be correlated with particular types of PA and SB (35). Undoubtedly, substance use has emerged as a prominent global public health concern, especially among adolescents, in industrialized and modernized societies for an extended period (36). Frequently misused substances include tobacco, alcohol, and cannabis (37). Studies consistently demonstrate that substance use tends to escalate during adolescence, with this pattern often initiating during this developmental stage and persisting into adulthood (38). In North America, substance use is prevalent among youths. Research by Joung et al. (39) highlights that in 2017, 56.8% of Canadian adolescents aged 15–19 consumed alcohol, compared to one-third of U.S. 12th graders. Additionally, 7.9% of Canadian adolescents smoked tobacco, while about 10% of U.S. 12th graders did the same (40). Drug consumption rates were 21.6% in Canada and 25% in the U.S. among youth (41). Similar trends are evident in other regions, including Australia (42), Thailand (43), and Spain (44). In China, substance use among youth is an increasing concern. Previous studies indicate that approximately 7.3% of Chinese children and adolescents aged 9 to 21 have reported drinking alcohol (45). A systematic review and meta-analysis found that the overall prevalence of smoking among Chinese youth is around 8.2% (46), with a significant increase in smoking rates observed among older adolescents (47).

Given that children and adolescents are in a crucial stage of growth and development, it is vital to prioritize their health behaviors during this time (48). Substance use during these formative years not only negatively affects health outcomes and lifestyle behaviors (49) but also predicts a variety of challenges for young people, including associations with delinquency and criminal behavior (50, 51). Given these significant risks, early prevention starting from primary school children is crucial, as this stage represents a formative period where foundational health behaviors are established, making it a critical time to intervene and mitigate future risks associated with smoking and alcohol use (52). While prior research has focused on adolescents, studies frequently highlight that the relationship between substance use and overall health is inversely related in this population (53). Additionally, sociodemographic factors combined with substance use can influence the 24-h movement behaviors of adolescents, such as increasing SB when alcohol and tobacco are used (54). Conversely, substance use is negatively associated with sleep quality, as demonstrated in a longitudinal study of U.S. adolescents from 1991 to 2014, where alcohol consumption was specifically linked to disrupted sleep patterns (55). Interestingly, previous research has suggested a potential correlation between alcohol use and higher levels of PA, possibly attributed to increased social interactions during sports events where alcohol is frequently consumed (56). Overall, the evidence suggests that while alcohol may encourage more PA, substance use generally promotes SB and impairs sleep, contributing to unhealthy 24-h movement behaviors in young people (57).

Nevertheless, most existing research exploring the links between socioeconomic factors, dietary intake, and substance use with sleep patterns, PA, and SB has been conducted in high-income Western countries (58, 59). This geographic concentration may limit the generalizability of findings, as socio-economic and cultural contexts vary significantly across different regions, potentially affecting sleep and activity patterns in diverse ways. This raises concerns about the applicability of these findings to low-and middle-income regions, particularly in less-developed countries in Asia, Africa, and South America, where cultural norms, legal frameworks, and social structures differ significantly. Recent empirical studies from China have broadened this research scope. Luo et al. (60) examined the relationship between adherence to the 24-HMG and health outcomes in older adult Chinese adults. Following this, Luo et al. (60) investigated how compliance with the 24-HMG affected depression and anxiety in Chinese adolescents. Furthering this line of inquiry, Luo (2024) (61) found that following the Sleep and Physical Activity recommendations of the 24-HMG significantly reduced the risk of visual impairments in non-obese adolescents. Despite these contributions, research in this area remains inadequate given the complexity of the issues involved. More studies are essential to deepen our understanding of how adherence to the 24-HMG affects health outcomes across diverse populations and age groups, especially in varied socioeconomic and cultural settings. Therefore, this study aims to explore the association between adherence to the 24-HMG (Combination of physical activity, screen time and sleep) and smoking and drinking behaviors among Chinese children and adolescents.

## 2 Methods

### 2.1 Participants and study design

In March 2021, a large-scale survey was conducted in collaboration with the Municipal Education Commission across primary and middle schools in Shenzhen, China. The survey encompassed students from all local public primary and secondary schools distributed throughout Shenzhen’s districts. Prior studies on PA among Chinese children and adolescents indicated that those aged 10 years and older possess adequate reading comprehension and can independently complete self-report questionnaires (62). Only students from grade 5 and higher were included as eligible participants due to developmental differences between younger and older students (63); those in grade 5 and above typically possess the cognitive and emotional maturity required to comprehend and accurately respond to the questionnaire. Therefore, the study population comprised students from upper primary (Mean ± SD: 11.5 ± 0.00), junior middle (Mean ± SD: 13.3 ± 0.00), and senior high schools (Mean ± SD: 16.0 ± 0.01).

Participants and their guardians received a written document detailing the study’s objectives. The survey aimed to gather data on physical and mental health outcomes, movement behaviors, and sociodemographic factors. Each student voluntarily completed the survey within approximately 20 min (Median: 18.27 min); all data were collected anonymously. Students who agreed to participate filled out the online questionnaire independently in the school’s computer room during a designated class period, under the supervision of a teacher who assisted as needed. The questionnaire was hosted on a widely used Chinese online platform^1^. An electronic informed consent form was displayed before participants could access the main survey questions. Only those who consented proceeded to the formal data collection stages. The survey protocol was approved by the Shenzhen University Research Committee (No. 2020005) and the participating schools.

A total of 78,428 participants initially completed the questionnaire. However, after excluding entries from participants who completed the questionnaire in less than 5 min or responded incorrectly to a key option item and considering the availability of variables required for the current analysis, the final sample size was reduced to 67,281 students.

### 2.2 Measures

Sleep duration was assessed using the Pittsburgh Sleep Quality Index (PSQI), which asks participants, “How many hours did you actually sleep at night in the past month?” The Chinese version of the PSQI has been validated for use among Chinese adolescents (64). Screen time was evaluated with items from the Health Behavior of School-aged Children (HBSC) survey, which includes various screen activities such as TV/movies, video games, and other leisure screen use over the past 7 days. Average daily screen time was calculated using the formula: ([total weekday screen time × 5] + [total weekend screen time × 2])/7. PA was measured with a single item adapted from the HBSC survey, asking, “How many days did you engage in MVPA for at least 60 min on weekdays over the past week?” with responses ranging from 0 (none) to 7 (every day). This measure has demonstrated satisfactory reliability among Chinese adolescents (65) and is extensively used in studies with Chinese children and adolescents (66, 67). According to the Canadian 24-Hour Movement Guidelines for Children and Youth (37), achieving 7 days of at least 60 min of MVPA is considered meeting the PA guidelines. Limiting screen time to no more than 2 ho per day meets the screen time (ST) guidelines. For sleep, 9–11 h per night for children (ages 5–12) and 8–10 h for adolescents (ages 13–17) are the benchmarks for guideline adherence. Compliance with these guidelines is assessed either by the total number of guidelines met (none, one, two, or three) or by specific combinations such as PA only, ST only, sleep only, combinations of two, or all three guidelines.

The survey collected comprehensive demographic and personal information, including sex (male/female), educational level by grade (primary school/junior middle school/senior middle school), self-reported height in centimeters, and weight in kilograms. Additional data included the number of siblings (only child or not), family structure (both parents or single parent), parental education level (ranging from junior middle school or below to master’s degree or above), ethnicity (Han or minority), and family socioeconomic status (SES). The geographic scope encompassed nine districts and 135 schools in total. Participants’ weight status was assessed using the body mass index (BMI), calculated from self-reported height and weight, and classified according to China’s normative reference data (68) into categories of normal weight, overweight, and obese. SES was evaluated using an adapted version of the MacArthur Scale of Subjective Social Status, which features a 10-rung ladder, with higher rungs indicating better socioeconomic status (69). To ensure robust statistical analysis, previous studies have controlled these variables to minimize confounding biases (70, 71).

Substance use behavior was assessed using items adapted from the Youth Risk Behavior Survey (72). The evaluation encompassed three distinct types of substance use behavior: whether participants had ever tried cigarette smoking, even if just one or two puffs; whether they had consumed at least one alcoholic drink beyond a few sips; and whether they had experienced drunkenness after consuming alcohol. Respondents were provided with the response options “Yes” or “No.” These items were chosen based partly on pilot feedback and our previous research endeavors (72, 73).

### 2.3 Statistical analysis

Statistical analyses were conducted using STATA BE 17.0 (College Station, Texas, USA), employing descriptive statistics to outline sample characteristics. Given the nested data structure across different layers—district (level 3), school (level 2), and individual (level 1), informed by the sampling strategy—a three-level mixed multilevel effect model was utilized to evaluate the associations between adherence to the 24-HMG and substance use. Substance use, the dependent variable, was treated as an ordinal variable for further analysis. A series of ordinal logistic regression models were then developed to examine these associations, controlling for all mentioned sociodemographic variables (e.g., SES, sex). Specifically, models 1 and 2 evaluated the number of guidelines met and specific combinations met, respectively, with “none” as the reference category, examining impacts on smoking and drinking. An interaction effect between grade group and adherence to the 24-HMG on substance use was detected, prompting stratified analyses by grade group, while controlling for other sociodemographic factors. Results were expressed as odds ratios (ORs) with 95% confidence intervals (CIs), considering *p*-values less than 0.05 as statistically significant.

## 3 Results

Table 1 presents the socio-demographic characteristics of the study participants. The sample was nearly evenly split between boys (51.9%) and girls (48%), with most participants from primary and junior middle schools. While 54.6% met two guidelines, only 1.7% met all three, and 28.7% met none. Compliance was highest for screen time (61.0%) but low for physical activity (9.3%) and sleep (19.6%). Smoking and alcohol use were rare, with 97.51% never smoking and 86.7% never alcohol use. The mean subjective family socioeconomic status was 5.0 (SD = 1.7).

**Table 1:** Sample characteristics.

Categorical variables		*n*	Mean ± SD/%
Sex	Boys (13.0 ± 0.01)	34,909	51.9
	Girls (13.1 ± 0.01)	32,372	48.1
Grade	Primary school (11.5 ± 0.00)	27,954	41.5
	Junior middle school (13.3 ± 0.00)	27,124	40.3
	High school (16.0 ± 0.01)	12,203	18.1
Weight status	Normal	45,817	68.1
	Overweight	9,051	13.5
	Obese	12,413	18.4
Siblings	Only child	17,354	25.8
	Non-only child	49,927	74.2
Family structure	Both parents	62,836	93.4
	Single	4,445	6.6
Paternal education	Junior middle school or below	14,619	21.7
	High school or equivalent	18,159	27
	Bachelor or equivalent	26,030	38.7
	Master or above	2,796	4.2
	Unclear	5,677	8.4
Maternal education	Junior middle school or below	17,617	26.2
	High school or equivalent	18,706	27.8
	Bachelor or equivalent	23,922	35.6
	Master or above	1,635	2.4
	Unclear	5,401	8
Nationality	Han	65,027	96.6
	Minority	2,254	3.4
Number of guidelines met	0	19,277	28.7
	1	36,712	54.6
	2	10,174	15.1
	3	1,118	1.7
Specific combinations of guidelines met	None	19,277	28.7
	Sleep only	4,611	6.9
	Screen time only	30,336	45.1
	MVPA only	1,765	2.6
	Sleep and screen time	6,823	10.1
	Sleep and MVPA	603	0.9
	Screen time and MVPA	2,748	4.1
	All three	1,118	1.7
Smoking	Never	65,609	97.51
	Rarely	797	1.18
	Occasionally	473	0.7
	Frequently	149	0.22
	Almost daily	253	0.38
Alcohol use	Never	58,333	86.7
	Rarely	6,196	9.21
	Occasionally	2,245	3.34
	Frequently	306	0.45
	Almost daily	201	0.3
Continuous variables		Mean	SD
Age (years)		13	1.8
Subjective family socioeconomic status		5	1.7

SD, standard deviation. Subjective family socioeconomic status was scaled from 0 to 10. MVPA, moderate to vigorous physical activity.

Table 2 highlights the associations between adherence to 24-HMG and smoking behavior. Participants who met none of the guidelines were more likely to smoke (OR = 1.62, *p* = 0.037). Adherence to one or two guidelines showed no significant effect on smoking likelihood.

**Table 2:** The association between adherence to 24-h movement guidelines and smoke.

	OR	95%CI		*p*
Number of guidelines met				
0	1.62	1.03	2.56	0.04
1	0.96	0.61	1.52	0.87
2	0.84	0.52	1.34	0.46
3	Reference group			
Combination of guidelines met				
None	1.58	1.00	2.49	0.05
Sleep only	1.11	0.68	1.79	0.68
Screen only	0.80	0.50	1.26	0.33
MVPA only	2.69	1.65	4.38	0.00
Sleep + screen time	0.55	0.33	0.91	0.02
Sleep + MVPA	1.52	0.81	2.85	0.19
Screen time + MVPA	1.30	0.78	2.15	0.31
All	Reference group			

OR, Odds Ratio; CI, Confidence Interval; Std. err, standard error; MVPA, moderate to vigorous physical activity; z, *z* value; p, *p-*value.

Regarding specific combinations of guidelines, adherence to only the screen time recommendation was not associated with lower smoking rates (OR = 0.80 [95% CI: 0.50, 1.26], *p* = 0.68), nor was adherence to only the sleep recommendation (OR = 1.11 [95% CI: 0.68, 1.79], *p* = 0.68). However, adherence to only the MVPA guideline significantly increased the likelihood of smoking (OR = 2.69 [95% CI: 1.65, 4.38], *p* < 0.001). Combinations of sleep and screen time recommendations were associated with a lower likelihood of smoking (OR = 0.55 [95% CI: 0.33, 0.91], *p* = 0.02), while the combination of screen and MVPA guidelines did not reach statistical significance (OR = 1.30 [95% CI: 0.78, 2.15], *p* = 0.31). Participants meeting none of the guidelines also demonstrated an increased likelihood of smoking (OR = 1.58 [95% CI: 1.00, 2.49], *p* = 0.05).

Table 3 illustrates an inverse relationship between adherence to 24-HMG and the likelihood of drinking. Participants who met none of the guidelines had the highest likelihood of alcohol use (OR = 2.07, *p* < 0.001), followed by those who met one guideline (OR = 1.40, *p* = 0.006), while meeting two guidelines showed a non-significant effect (OR = 1.22, *p* = 0.106).

**Table 3:** The association between adhering to 24-h movement guidelines and drink.

	OR	95%CI		*P*
Number of guidelines met				
0	2.07	1.63	2.63	0.00
1	1.40	1.10	1.77	0.01
2	1.22	0.96	1.56	0.10
Combination of guidelines met				
None	2.04	1.61	2.59	0.00
Sleep only	1.37	1.06	1.76	0.02
Screen only	1.31	1.03	1.66	0.03
MVPA only	2.85	2.19	3.70	0.00
Sleep + Screen time	0.97	0.75	1.24	0.78
Sleep + MVPA	2.00	1.44	2.78	0.00
Screen + MVPA	1.68	1.29	2.18	0.00
All	Reference group			

OR, odds ratio; CI, confidence interval. Std. err, standard error; MVPA, moderate to vigorous physical activity; z, *z* value; *p, p-*value.

Specific findings include adherence to only the screen time guideline (OR = 1.31, *p* = 0.028), only the MVPA guideline (OR = 2.85, *p* < 0.001), and only the sleep guideline (OR = 1.37, *p* = 0.015) all showing significant associations. Combinations of guidelines showed varied results: sleep and screen time (OR = 0.97, *p* = 0.783), screen time and MVPA (OR = 1.68, *p* < 0.001), and sleep and MVPA (OR = 2.0, *p* < 0.001) were also examined for their association with drinking behaviors.

## 4 Discussion

This study explored the relationship between adherence to the 24-HMG and the incidence of smoking and alcohol use among children and adolescents. The findings indicated that over half of the participants adhered to at least two of the movement guidelines, although adherence to the PA guidelines was particularly low. A significant association was found between adherence to these guidelines and a reduction in smoking and alcohol consumption. These results highlight a negative correlation between the extent of guideline adherence and the likelihood of engaging in harmful behaviors. This study emphasizes the potential role of routine adherence to movement guidelines in promoting healthier lifestyles among children and adolescents.

This study explored the complex relationship between adherence to the 24-HMG and smoking behavior among children and adolescents, highlighting the differential impacts of various components on smoking levels. Strikingly, only 1.7% of participants adhered to all three guidelines, reflecting the challenge of full compliance (OR = 0.84, *p* = 0.459). This low compliance rate suggests that comprehensive engagement with health behaviors may be associated with reduced smoking behaviors. Specifically, adherence to the MVPA guidelines showed a significant negative correlation with smoking (OR = 2.69, *p* < 0.001), consistent with the findings of Gismero-González et al. (74), who reported that vigorous exercise notably deters smoking, especially among beginners. Our findings reinforce this correlation, particularly in participants meeting both MVPA and sleep + MVPA guidelines, suggesting that the emotional benefits of physical exercise and its role in enhancing health consciousness could be key factors (75). These results align with previous research and underscore the importance of integrated health behaviors in reducing smoking prevalence among children and adolescents.

Conversely, adherence to screen time guidelines alone did not significantly impact smoking rates (OR = 0.8, *p* = 0.68), indicating that increased screen time may not directly influence smoking but could contribute to behaviors such as sedentariness, which are linked to smoking susceptibility (76). Similarly, adherence to sleep guidelines alone showed no significant reduction in smoking (OR = 1.11, *p* = 0.68), which contrasts with Wheaton’s (77) findings on the adverse effects of sleep deprivation on health behaviors. This study underscores the synergistic effects of adhering to multiple guidelines, illustrating that a holistic approach to health behaviors—such as combining adherence to sleep and screen time guidelines—can more effectively reduce smoking (OR = 0.55, *p* = 0.02). This finding supports the work of Barua et al. (78), who advocate for comprehensive health interventions rather than isolated behavioral changes to decrease youth smoking. An integrated strategy may help bridge the physical and mental aspects of health, guiding children and adolescents toward a healthier lifestyle and long-term avoidance of smoking.

This study highlights the complex relationship between lifestyle choices and adolescent health outcomes, particularly in relation to drinking behaviors. The findings reveal an inverse correlation between adherence to the 24-HMG and the prevalence of alcohol consumption among children and adolescents. Notably, adherence to MVPA guidelines alone showed the strongest deterrent effect on drinking behaviors (OR = 2.85, *p* < 0.001). However, not all 24-HMG components were associated with drinking. For instance, adherence to sleep and screen time guidelines combined was not linked to reduced drinking. In contrast, significant inverse correlations were found for PA alone, as well as for combinations like sleep + PA and screen time + PA, suggesting that physically active adolescents tend to focus on fitness, which correlates with lower engagement in harmful behaviors like alcohol consumption (79). PA is associated with promoting a healthier lifestyle and acts as a preventive measure against negative behaviors, including alcohol use, especially among infrequent drinkers (80). However, Liu et al. (2015) (81) highlighted that while PA can boost self-esteem and social connections, it might paradoxically increase alcohol consumption due to greater social opportunities (82), indicating that adolescent drinking behaviors are influenced by broader social contexts. Additionally, adherence to screen time guidelines also contributed to reduced drinking rates (OR = 1.31, *p* = 0.028), likely due to reduced exposure to alcohol-related advertising (83). Furthermore, sufficient sleep was linked to lower alcohol consumption (OR = 1.37, *p* = 0.015), as proper rest is associated with reduced stress and improved decision-making, which lowers the likelihood of drinking to alleviate stress (84). The combined adherence to sleep and PA guidelines significantly reinforced this inverse relationship (OR = 2.85, *p* < 0.001), supporting Bhochhibhoya and Branscum’s (85) assertion that an integrated behavioral strategy can effectively decrease the propensity for alcohol and drug use among children and adolescents. This study provides several implications for enhancing adherence to the 24-HMG among Chinese children and adolescents, with a particular focus on public health policies and educational strategies. A key takeaway is the pivotal role of school and family environments in fostering a healthy lifestyle (86–88). Schools can optimize their facilities to encourage PA, incorporate proper sleep schedules, and establish norms for screen use within their educational programs. These initiatives align with findings from previous studies that emphasize the importance of school-based interventions in fostering healthy behaviors among children and adolescents (89). Additionally, government policies could be instrumental in establishing and enforcing standards for physical education in school curricula. Such policies should not only prioritize PA as a fundamental component of quality education but also provide structured alternatives to minimize unsupervised screen time, supporting earlier research that links reduced screen time with better health outcomes (90). Moreover, regular family health awareness seminars could improve the effectiveness of guideline implementation, as family-based interventions are crucial for promoting children’s adherence to healthy behaviors (91). Given the cultural context of China, where the education system and family structures often emphasize academic performance, creating significant competitive pressure on teenagers (92, 93). It is essential to integrate mental health support with physical health measures and substance use management. Achieving these goals will require coordinated efforts among schools, families, and government entities to significantly enhance the public health status of China’s youth and reduce the prevalence of harmful behaviors such as smoking and drinking. While this study provides valuable insights into the relationship between adherence to lifestyle guidelines and youth behavior in China, future research should further explore broader environmental factors. Such research would help clarify the underlying influences on these behaviors and more effectively guide public health interventions.

This study demonstrates both strengths and limitations. One significant strength lies in its comprehensive approach, encompassing a wide array of lifestyle factors and utilizing diverse indicators to thoroughly evaluate the cumulative impact of health behaviors. Furthermore, the study benefits from a large sample size, which contributes to robust data sets, facilitating a more nuanced understanding of correlations between indicators and bolstering the generalizability of the findings within the Chinese context. However, a primary limitation stems from the reliance on self-reported data, which introduces the possibility of response bias. Participants may tend to underreport behaviors perceived as negative, such as smoking and drinking, potentially biasing the results. Additionally, the study does not delve deeply into the causal relationships between various external factors and health behaviors. For example, the influences of socioeconomic status and educational environments on adolescent behavior were not thoroughly explored.

## 5 Conclusion

This study investigates the influence of adhering to 24-HMG on smoking and drinking among children and adolescents. The results suggest that while more than half of the participants adhered to at least two of these guidelines, compliance with PA recommendations was notably lower. Importantly, the data unveiled a distinct correlation between adherence to these guidelines and a decrease in smoking and alcohol consumption. This underscores the inverse link between following the guidelines and participating in harmful behaviors, thereby emphasizing the study’s aim to demonstrate how daily habits can steer adolescents toward healthier lifestyles.

## Data Availability

The datasets presented in this study can be found in online repositories. The names of the repository/repositories and accession number(s) can be found in the article/supplementary material.

## Footnotes

^1^ https://www.wjx.cn/
